# Association analysis of cigarette smoking with onset of primary open-angle glaucoma and glaucoma-related biometric parameters

**DOI:** 10.1186/1471-2415-12-59

**Published:** 2012-11-27

**Authors:** Degui Wang, Yuqiang Huang, Chukai Huang, Pengfei Wu, Jianwei Lin, Yuqian Zheng, Yi Peng, Yuanbo Liang, Jian-Huan Chen, Mingzhi Zhang

**Affiliations:** 1 Joint Shantou International Eye Center, Shantou University & the Chinese University of Hong Kong, Shantou, China; 2 Department of Ophthalmology and Visual Sciences, The Chinese University of Hong Kong, Hong Kong, China

**Keywords:** Primary open angle glaucoma, Cigarette smoking, Central corneal thickness, Vertical cup-to-disc ratio, Intraocular pressure

## Abstract

**Background:**

To date, studies on the role played by cigarette smoking in primary open-angle glaucoma (POAG) remains controversial. The current study evaluated cigarette smoking as a risk factor of POAG and its relationships with vertical cup-to-disc ratio (VCDR), central corneal thickness (CCT) and intraocular pressure (IOP) in a Chinese cohort.

**Methods:**

In a total of 248 unrelated individuals including 30 juvenile-onset POAG (JOAG), 92 adult-onset POAG (AOAG) and 126 sex-matched senile cataract controls, underwent comprehensive ophthalmic examination. Their smoking was obtained and documented by questionnaire. Association of cigarette smoking with POAG was performed using logistic regression controlled for age and sex. Effects of cigarette smoking on VCDR, IOP and CCT were analyzed with multiple linear regression.

**Results:**

In either JOAG or AOAG, no association of cigarette smoking was found with disease onset (*P* = 0.692 and 0.925 respectively). In controls and JOAG, no significant effects of smoking were found on VCDR, IOP or CCT (all *P* > 0.05). Smoking was found to be correlated with decreased CCT in AOAG and combined POAG (JOAG + AOAG) (*P* = 0.009 and 0.003), but no association with VCDR or IOP was observed (*P* > 0.05).

**Conclusions:**

Although cigarette smoking was not found to be risk factor for onset of POAG, it was correlated with CCT in AOAG, and thus might still play a role in the disease course, especially for AOAG.

## Background

Primary open-angle glaucoma (POAG) is a group of disorders characterized by loss of retinal ganglion cells associated with optic nerve degeneration and visual field loss. It’s the second leading cause of visual loss and blindness worldwide, and affected 60 million people [1]. POAG is associated with many clinical features, including vertical cup-to-disc ratio (VCDR), central corneal thickness (CCT) and intraocular pressure (IOP). According to age of disease onset, there are two categories of POAG: adult-onset POAG (AOAG, disease onset after 40 years) and juvenile-onset POAG (JOAG, disease onset between 3 and 40 years) [2]. Reported risk factors of POAG include cigarette smoking, hypertension, diabetes, and family history [3-5]. Although existing studies have reported the association between cigarette smoking and POAG [6,7], the role of cigarette smoking as a risk factor of POAG remains controversial [8,9]. And most of these studies focused on AOAG. Especially, there are limited data of these studies in the Chinese population. In this study we investigated the association of cigarette smoking with POAG, and its relationship with VCDR, CCT and IOP in a Southern Chinese cohort.

## Methods

### Patient recruitment and clinical information

The study subjects were unrelated, and included 122 POAG patients and 126 controls recruited at the Joint Shantou International Eye Center in Shantou, China from March, 2008 to November, 2011 (Table 1). Both POAG patients and controls received comprehensive ophthalmic examination. Their highest IOP of both eyes before medication, maximum VCDR of both eyes, and mean CCT were documented when they were first presented to the clinic. The highest IOP was measured using Goldman Ocular tonometry. VCDR and CCT were measured using a standard protocol. Before measuring, a single drop of proparacaine 1% (Alcaine, Alcon Laboratories, Ft. Worth TX) was placed in the eye. VCDR was measured by an experienced glaucoma specialist, and then confirmed by another experienced glaucoma specialist. IOP was measured by an experienced glaucoma specialist using Goldmann applanation tonometry, and recorded as the mean of three measurements. CCT was measured ultrasonically (IOPac 20 Mhz Pachymeter, Heidelberg Engineering, Germany). A total of 10 measurements were made for each eye, with additional measurements obtained if the standard deviation exceeded 10 microns. Visual field function was assessed using the Glaucoma Hemifield test (Humphrey automated perimetry, Carl Zeiss Meditec, Inc., Germany). Cigarette smoking status was obtained by questionnaire (Additional file 1), and active smoking was defined as smoking at least 5 cigarettes per day for in the past one or more years [3].

**Table 1:** Demographic features of the study subjects

	**N**	**Female**	**Age (Year)**		**VCDR**		**IOP (mmHg)**		**CCT (μM)**	
			**Mean**	**SD**	**Mean**	**SD**	**Mean**	**SD**	**Mean**	**SD**
Control	126	29	69.5	8.9	0.32	0.06	13.3	2.6	541.7	36.1
JOAG	30	6	25	6	0.86	0.17	34.1	11.4	542.6	21.9
AOAG	92	14	59.9	12.1	0.84	0.14	31.5	10.9	533.9	36.4

The diagnosis of POAG was based on the following inclusion criteria: (1) gonioscopically open anterior chamber angle, Shaffer grade III or IV; (2) characteristic optic disc damage and/or typical visual field loss; (3) highest IOP > 21 mmHg; (4) exclusion of secondary causes, e.g., trauma, uveitis, steroid-induced or exfoliation glaucoma. Juvenile-onset POAG (JOAG) was recruited based on age of disease onset between 3 and 40 years, and adult-onset POAG (AOAG) based on disease onset after 40 years. The control subjects were recruited from senile cataract surgical inpatients aged 50 and older without family history of glaucoma. All of the controls have IOP < 21 mmHg, VCDR < 0.5, and no sign of visual field loss. Eyes met the following criteria were excluded: any history or symptom of keratopathy, Marfan’s syndrome, ocular trauma, ocular surgery in prior to the recruitment, macular epiretinal membrane, macular edema, macular hemorrhage, retinal detachment, or severe cataract affecting the results of examination.

This study was approved by the Ethics Committee of Joint Shantou International Eye Center and was conducted in accordance with the Declaration of Helsinki. Written consent was obtained from each participating subject after explanation of the nature of the study.

### Statistical analysis

Association and interaction analysis was performed using multiple regression implemented by the R statistical Language version 2.15.0. Association between cigarette smoking and disease was assessed in both JOAG and AOAG by logistic regression controlled age and sex. Multiple linear regression was used to analyze effects of cigarette smoking on VCDR, IOP and CCT. Two-factor interaction between smoking and age/sex was evaluated in both logistic and linear regression.

## Results

### Patients and clinical data

Their clinical features were shown in Table 1. Among POAG patients, 30 were diagnosed as juvenile-onset POAG (JOAG), 92 as adult-onset POAG (AOAG). Ocular hypertension and signs of optic disc neuropathy were found in these POAG patients, but were absent in all 126 senile cataract controls.

### Relationship between cigarette smoking and disease onset of POAG

As shown in Table 2, in the current study no association of cigarette smoking was found with disease onset of JOAG after adjusted for age and sex (adjusted *P* = 0.692). Similarly, no association of cigarette smoking was found in disease onset of AOAG (adjusted *P* = 0.925). No interaction between smoking and age/sex was detected in association with the disease onset (data not shown).

**Table 2:** Association of cigarette smoking with JOAG and AOAG in the current cohort

	**Control**	**JOAG**			**AOAG**		
	**N (%)**	**N (%)**	**OR (95%CI)**	P*	**N (%)**	**OR (95%CI)**	P*
Non-smoker	55 (43.7)	14 (41.2)	4.33 (0.12-5.84 × 10^5^)	0.692	31 (33.7)	1.03 (0.52-2.08)	0.925
Smoker	71 (56.3)	16 (58.8)			61 (66.3)		

*P-values are adjusted to age and sex.

### Correlation between cigarette smoking and glaucoma-related biometric parameters

We further examined the effects of cigarette smoking on glaucoma-related risk factors. Multiple linear regression analysis of VCDR, CCT and IOP were shown in Tables 3, 4, and 5. In controls, no significant effects of smoking were found on VCDR, IOP or CCT (all *P* > 0.05). In JOAG, none of smoking, age and sex showed significant association with VCDR, IOP or CCT (all *P* > 0.05). In AOAG, smoking was found to be significantly correlated with CCT (β ± s.e. = −32.7 ± 11.9, *P* = 0.009), but not with VCDR or IOP (*P* = 0.418 and 0.111 respectively). Neither age nor sex was correlated with VCDR, IOP or CCT in AOAG (all *P* > 0.05). A significant correlation between smoking and CCT was observed in combined POAG (JOAG + POAG) (β ± s.e. = −26.1 ± 8.5 μm, *P* = 0.003). No interaction between smoking and age/sex was detected in association with the glaucoma-related biometric parameters (data not shown).

**Table 3:** **Multiple linear regression analysis of effects of smoking**, **age and female sex on vertical cup**-**to**-**disc ratio**

	**Controls**		**JOAG**		**AOAG**		**JOAG + AOAG**	
	**β (SE)**	***P***	**β (SE)**	***P***	**β (SE)**	***P***	**β (SE)**	***P***
Smoking (Yes)	-0.003 (0.016)	0.867	-0.101 (0.069)	0.156	-0.019 (0.035)	0.590	-0.031 (0.0290	0.294
Age (Year)	0.025 (0.017)	0.150	0.011 (0.081)	0.893	-0.053 (0.046)	0.247	-0.034 (0.038)	0.370
Female sex	-0.001 (0.001)	0.202	0.006 (0.004)	0.207	0.001 (0.001)	0.502	0.000 (0.001)	0.907

**Table 4:** **Multiple linear regression analysis of effects of smoking**, **age and female sex on intraocular pressure**

	**Controls**		**JOAG**		**AOAG**		**JOAG + AOAG**	
	**β (SE)**	***P***	**β (SE)**	***P***	**β (SE)**	***P***	**β (SE)**	***P***
Smoking (Yes)	-0.632 (0.555)	0.257	2.430 (4.679)	0.608	-3.885 (2.764)	0.163	-1.061 (2.197)	0.630
Age (Year)	-0.583 (0.658)	0.377	3.741 (5.520)	0.504	-4.100 (3.594)	0.257	-0.471 (2.859)	0.869
Female sex	-0.015 (0.027)	0.580	-0.055 (0.289)	0.851	-0.090 (0.098)	0.365	-0.071 (0.056)	0.206

**Table 5:** **Multiple linear regression analysis of effects of smoking**, **age and female sex on central corneal thickness**

	**Controls**		**JOAG**		**AOAG**		**JOAG + AOAG**	
	**β (SE)**	***P***	**β (SE)**	***P***	**β (SE)**	***P***	**β (SE)**	***P***
Smoking (Yes)	-1.3 (7.8)	0.865	-13.6 (11.8)	0.271	-32.7 (11.9)	0.009	-26.1 (8.5)	0.003
Age (Year)	-16.3 (9.2)	0.080	-4.7 (12.3)	0.709	-23.2 (14.9)	0.128	-11.3 (10.2)	0.272
Female sex	0.6 (0.4)	0.103	0.1 (0.8)	0.926	0.6 (0.5)	0.188	0.1 (0.2)	0.657

## Discussion

According to World Health Organization data, smoking has become a serious global public health problem. Among the approximately 1.3 billion smokers in the world, over 6 million die annually due to tobacco exposure, especially in [10]. Cigarette smoking is related to many eye diseases such as cataract, age-related macular degeneration [11-13]. Existing studies on the relationship between cigarette smoking and POAG remain controversial. Findings vary among ethnic groups and study design. And most of these studies focused on AOAG. There are limited data of these studies in the Chinese population. Recently in a cohort of African-American women, Wise L. A. et al. reported that smoking might be associated with increased risk of early-onset POAG [14]. Kang, J. H. et al. also reported cigarette smoking conferring risk to POAG [15]. Similar findings were also reported in other independent studies [16-18]. But some other studies reported no association between smoking and POAG. In a prospective follow-up study from 1980 and 1986, respectively, to 1996, the results showed neither current smokers nor ex-smokers were at greater risk for POAG than those who had never smoked, and heavier smoking did not increase the risk of POAG [8]. And in a systematic review, Richard et al. also reported that there was little evidence for the association between smoking and POAG [19]. Other independent studies also reported lack of such association [9,20]. In our Chinese cohort, smoking was not found to confer risk to disease onset in either JOAG or AOAG. Our study thus did not support cigarette smoking as a risk factor of POAG onset.

Apart from disease onset, it remains to be elucidated whether cigarette smoking is related to IOP, CCT and VCDR, which are glaucoma risk factors. In 2003, Yoshida M. et al. reported that cigarette smoking had a significantly positive association with the IOP in Japanese male individuals [21]. Lee A. J. et al. also reported similar association [22]. In the current study, although no significant association with IOP was observed, we found evidence that smoking could be correlated with decreased CCT in AOAG. As reported by previous studies, in individuals with thinner cornea, their IOP tends to be lower estimated [23-25]. In AOAG smokers, their IOP could possibly be lower estimated due to thinner CCT. In addition, the change of CCT in POAG may affect the disease course. A previous study on corneal thickness and functional damage in patients with ocular hypertension showed that patients with ocular hypertension plus thinner cornea had a greater risk of developing functional damage over time [26].

The exact reason for decreased corneal thickness in POAG smokers remains unclear. However, cigarette smoking may exert this effect through hypoxia and collagen in the cornea. Smoking has been reported to decrease oxygen and collagen production in tissues during wound healing [27,28]. Ocular hypertension causes damage to the cornea [29]. Smoking probably deteriorates ocular hypoxia caused by ocular hypertension [27], and consequently affects the biosynthesis of collagen and extracellular matrix turnover [30], which could be an explanation to the decreased corneal thickness.

In the current study, cigarette smoking was not found to be associated with disease onset of POAG. However, the association of smoking with decreased CCT in POAG suggested that more attention should be paid on CCT in the early stage of POAG in smokers to estimate more correct target IOP in order to better reduce the early lesion of optic nerve. Current findings thus warranted further study.

## Conclusions

In this study, although cigarette smoking was not found to be a risk factor for onset of POAG, it was correlated with CCT in AOAG, and thus might still play a role in the disease course, especially for AOAG.

## Competing interests

The authors declare that they have no competing interests.

## Authors’ contributions

DW carried out the questionnaire, participated in statistical analysis and drafted the manuscript. YH and CH carried out the clinical examination in patients and controls. YZ participated in study coordination. PW and JL provided support of database software. YP and YL assisted in the statistical analysis and discussion. J-HC participated in the study design and statistical analysis. MZ conceived the study, participated in the study design and clinical examination, and wrote the manuscript. All authors read and approved the final manuscript.

## Pre-publication history

The pre-publication history for this paper can be accessed here:

http://www.biomedcentral.com/1471-2415/12/59/prepub

## Supplementary Material

Additional file 1 Smoking Status Questionnaire. Click here for file

## References

[B1] Quigley HA Broman AT The number of people with glaucoma worldwide in 2010 and 2020 Br J Ophthalmol 2006 90 3 262 267 10.1136/bjo.2005.081224 16488940 PMC1856963

[B2] Rao KN Nagireddy S Chakrabarti S Complex genetic mechanisms in glaucoma: an overview Indian J Ophthalmol 2011 59 Suppl S31 S42 21150032 10.4103/0301-4738.73685 PMC3038510

[B3] Fan BJ Leung YF Wang N Lam SC Liu Y Tam OS Pang CP Genetic and environmental risk factors for primary open-angle glaucoma Chin Med J (Engl) 2004 117 5 706 710 15161538

[B4] Raymond V Molecular genetics of the glaucomas: mapping of the first five “GLC” loci Am J Hum Genet 1997 60 2 272 277 9012399 PMC1712410

[B5] Leske MC The epidemiology of open-angle glaucoma: a review Am J Epidemiol 1983 118 2 166 191 6349332 10.1093/oxfordjournals.aje.a113626

[B6] Cheng AC Pang CP Leung AT Chua JK Fan DS Lam DS The association between cigarette smoking and ocular diseases Hong Kong Med J 2000 6 2 195 202 10895144

[B7] Solberg Y Rosner M Belkin M The association between cigarette smoking and ocular diseases Surv Ophthalmol 1998 42 6 535 547 10.1016/S0039-6257(98)00002-2 9635902

[B8] Kang JH Pasquale LR Rosner BA Willett WC Egan KM Faberowski N Hankinson SE Prospective study of cigarette smoking and the risk of primary open-angle glaucoma Arch Ophthalmol 2003 121 12 1762 1768 10.1001/archopht.121.12.1762 14662597

[B9] Klein BE Klein R Ritter LL Relationship of drinking alcohol and smoking to prevalence of open-angle glaucoma. The Beaver Dam Eye Study Ophthalmology 1993 100 11 1609 1613 8233383 10.1016/s0161-6420(93)31429-6

[B10] Wipfli H Samet JM Global economic and health benefits of tobacco control: part 1 Clin Pharmacol Ther 2009 86 3 263 271 10.1038/clpt.2009.93 19536067

[B11] Thornton J Edwards R Mitchell P Harrison RA Buchan I Kelly SP Smoking and age-related macular degeneration: a review of association Eye (Lond) 2005 19 9 935 944 10.1038/sj.eye.6701978 16151432

[B12] Lois N Abdelkader E Reglitz K Garden C Ayres JG Environmental tobacco smoke exposure and eye disease Br J Ophthalmol 2008 92 10 1304 1310 10.1136/bjo.2008.141168 18658170

[B13] Cong R Zhou B Sun Q Gu H Tang N Wang B Smoking and the risk of age-related macular degeneration: a meta-analysis Ann Epidemiol 2008 18 8 647 656 10.1016/j.annepidem.2008.04.002 18652983

[B14] Wise LA Rosenberg L Radin RG Mattox C Yang EB Palmer JR Seddon JM A prospective study of diabetes, lifestyle factors, and glaucoma among African-American women Ann Epidemiol 2011 21 6 430 439 10.1016/j.annepidem.2011.03.006 21549278 PMC3091261

[B15] Kang JH Wiggs JL Rosner BA Haines J Abdrabou W Pasquale LR Endothelial nitric oxide synthase gene variants and primary open-angle glaucoma: interactions with hypertension, alcohol intake, and cigarette smoking Arch Ophthalmol 2011 129 6 773 780 10.1001/archophthalmol.2011.118 21670344 PMC3337676

[B16] Mehra KS Roy PN Khare BB Tobacco smoking and glaucoma Ann Ophthalmol 1976 8 4 462 464 1267318

[B17] Wilson MR Hertzmark E Walker AM Childs-Shaw K Epstein DL A case–control study of risk factors in open angle glaucoma Arch Ophthalmol 1987 105 8 1066 1071 10.1001/archopht.1987.01060080068030 3632414

[B18] Bonovas S Filioussi K Tsantes A Peponis V Epidemiological association between cigarette smoking and primary open-angle glaucoma: a meta-analysis Public Health 2004 118 4 256 261 10.1016/j.puhe.2003.09.009 15121434

[B19] Edwards R Thornton J Ajit R Harrison RA Kelly SP Cigarette smoking and primary open angle glaucoma: a systematic review J Glaucoma 2008 17 7 558 566 10.1097/IJG.0b013e31815f530c 18854733

[B20] Ponte F Giuffre G Giammanco R Dardanoni G Risk factors of ocular hypertension and glaucoma. The casteldaccia Eye study Doc Ophthalmol 1994 85 3 203 210 10.1007/BF01664928 7924848

[B21] Yoshida M Ishikawa M Kokaze A Sekine Y Matsunaga N Uchida Y Takashima Y Association of life-style with intraocular pressure in middle-aged and older Japanese residents Jpn J Ophthalmol 2003 47 2 191 198 10.1016/S0021-5155(02)00666-4 12738554

[B22] Lee AJ Rochtchina E Wang JJ Healey PR Mitchell P Does smoking affect intraocular pressure? findings from the blue mountains Eye study J Glaucoma 2003 12 3 209 212 10.1097/00061198-200306000-00005 12782837

[B23] Avitabile T Longo A Rocca D Amato R Gagliano C Castaing M The influence of refractive errors on IOP measurement by rebound tonometry (ICare) and Goldmann applanation tonometry Graefes Arch Clin Exp Ophthalmol 2010 248 4 585 591 10.1007/s00417-009-1176-5 19727794

[B24] Ehlers N On corneal thickness and intraocular pressure. II. A clinical study on the thickness of the corneal stroma in glaucomatous eyes Acta Ophthalmol (Copenh) 1970 48 6 1107 1112 5537252 10.1111/j.1755-3768.1970.tb06591.x

[B25] Kohlhaas M Boehm AG Spoerl E Pursten A Grein HJ Pillunat LE Effect of central corneal thickness, corneal curvature, and axial length on applanation tonometry Arch Ophthalmol 2006 124 4 471 476 10.1001/archopht.124.4.471 16606871

[B26] Zeppieri M Brusini P Miglior S Corneal thickness and functional damage in patients with ocular hypertension Eur J Ophthalmol 2005 15 2 196 201 15812759 10.1177/112067210501500203

[B27] Jensen JA Goodson WH Hopf HW Hunt TK Cigarette smoking decreases tissue oxygen Arch Surg 1991 126 9 1131 1134 10.1001/archsurg.1991.01410330093013 1929845

[B28] Jorgensen LN Kallehave F Christensen E Siana JE Gottrup F Less collagen production in smokers Surgery 1998 123 4 450 455 10.1016/S0039-6060(98)70167-9 9551072

[B29] Melamed S Ben-Sira I Ben-Shaul Y Corneal endothelial changes under induced intraocular pressure elevation: a scanning and transmission electron microscopic study in rabbits Br J Ophthalmol 1980 64 3 164 169 10.1136/bjo.64.3.164 7387948 PMC1039380

[B30] Knuutinen A Kokkonen N Risteli J Vahakangas K Kallioinen M Salo T Sorsa T Oikarinen A Smoking affects collagen synthesis and extracellular matrix turnover in human skin Br J Dermatol 2002 146 4 588 594 10.1046/j.1365-2133.2002.04694.x 11966688

