# Supplementary material for: Association analysis of cigarette smoking with onset of primary open-angle glaucoma and glaucoma-related biometric parameters
Source: BMC Ophthalmol. 2012 Nov 27;12:59. doi: 10.1186/1471-2415-12-59 (PMC3549870; doi:10.1186/1471-2415-12-59)
Supplement: Additional file 1 — Smoking Status Questionnaire. [file 1471-2415-12-59-S1.doc]

**Smoking Status Questionnaire**

Q1. Have you ever smoked in your entire life?

A. Never B. Yes, but have quitted C. Yes, currently smoke

(If you choose B. please go to Q2 and Q3; if you choose C. please go to Q4)

Q2. When did you quit smoking?

A. Less than 6 months ago B. 6~12 months ago C. more than 1 year ago

Q3. How long had you smoked before you quitted?

A. less than 6 months B. 6~12 months C. more than 1 year

Q4, How often do you smoke?

A. few B. sometimes C. everyday

(If you choose C, please answer Q5)

Q5, How many cigarettes do you smoke per day?

A. 1~4 B. 5~14 C. 15~24 D. more than 24

(If you choose B, C, or D, please answer Q6)

Q6. How long have you been smoking regularly?

A. less than 6 months B. 6~12 months C. more than 1 year

Notes:

Active Smoker: smoke at least 5 cigarettes per day for more than 1 year

Non-smoker: never smoke

Ex-smoker: have smoking before, now quitted at least 1 year ago

Others have been excluded
